# Modification of cleaning product formulations could improve indoor air quality

**DOI:** 10.1111/ina.13021

**Published:** 2022-03-28

**Authors:** Nicola Carslaw, David Shaw

**Affiliations:** ^1^ Department of Environment and Geography University of York York UK

**Keywords:** cleaning product formulations, fragrances, indoor air chemistry, indoor air modeling, indoor oxidation

## Abstract

Cleaning products contain numerous individual chemicals, which can be liberated on use. These species can react in air to form new chemical species, some of which are harmful to health. This paper uses a detailed chemical model for indoor air chemistry, to understand the chemical reactions that can occur following cleaning, assuming cleaning products with different proportions of limonene, α‐pinene, and β‐pinene are used. The tests included the pure compounds, 50:50 mixtures and mixtures in proportion to the rates of reaction with ozone and the hydroxyl radical. For the 3 h following cleaning, pure α‐pinene was most efficient at producing particles, pure limonene for nitrated organic material, and a 50:50 mixture of β‐pinene and limonene for formaldehyde, leading to enhancements of 1.1 μg/m^3^, 400 ppt, and 1.8 ppb, respectively, compared to no cleaning. Cleaning in the afternoon enhanced concentrations of secondary pollutants for all the mixtures, owing to higher outdoor and hence indoor ozone compared to the morning. These enhancements in concentrations lasted several hours, despite the cleaning emissions only lasting for 10 min. Doubling the air exchange rate enhanced concentrations of formaldehyde and particulate matter by ~15% while reducing that of nitrated organic material by 13%. Changing product formulations has the potential to change the resulting indoor air quality and consequently, impacts on health.

**Practical Implications**

Changing the formulation of terpene‐based cleaning products leads to changes in the resulting secondary pollutant composition that is observed. Some of the secondary pollutants that are formed following the use of such products can be harmful to human health, such as particulate matter and formaldehyde. The results from this study suggest that it is possible to vary the proportion of terpenes within cleaning and other consumer products to produce lower concentrations of potentially harmful pollutants indoors.

## 1 INTRODUCTION

Cleaning products are now ubiquitous in our lives. They are available for a wide range of applications and exist in numerous formulations, containing components including terpenes, bleach (chlorine), amines, quaternary ammonium compounds, aldehydes, hydrochloric acid, and sodium hydroxide.^1^ They are widely perceived to have been at least partially responsible for improvements in hygiene and concomitant decrease in poor hygiene‐related diseases over recent years, although Nazaroff and Weschler^2^ note that there is little scientific evidence that supports the efficacy of cleaning with such products. Maybe because of this perception, the average US adult spends 20–30 min a day cleaning their home, including the use of cleaning fluids.^3^

Cleaning products are complex and contain numerous components. They typically contain a product base mixture and potentially several hundred unique fragrance products.^4^, ^5^ The fragrance is the key selling point for many consumers, and the fragrance industry is constantly producing new and complex mixtures of natural and synthetic ingredients to gain commercial success.^6^ The exact composition of fragranced products is often hidden for confidentiality/commercial reasons. For instance, during an investigation of the composition of 25 commonly used products in the United States, 133 volatile organic compounds (VOCs) were identified, but only 1 of these was listed on a product label.^4^

Exposure to fragrances can elicit various adverse health effects in some people^4^ and many countries have regulations in place to address their use in commercial products. For instance, in Europe, manufacturers are bound by Regulation (EC) 648/2004 on detergents, which states that 26 specified fragrance allergens must be listed on the detergent container if they exceed 0.01% by concentration in a particular product and if they are added as a single substance rather than as part of a natural extract.^7^ All other fragrances, or any of the 26 specified above if present at concentrations below 0.01% of the total, can be listed as “perfume” or some similar wording.^7^

Terpenoid species are common components of cleaning compounds and are used as active ingredients or fragrances in many of them.^5^, ^8^ Wieck et al.^7^ carried out a survey of 131 households in Northern Germany in 2015 and analyzed the ingredients of 1447 detergents. Limonene, linalool, and hexyl cinnamal were the most frequent fragrance allergens listed within many of the products they tested. They also found that there were between 24 and 270 individual chemicals within the detergents, and that around a third to half of each category of detergent contained fragrance allergens. In a study in the United States, Steinemann et al.^4^ investigated 25 commonly used fragranced consumer products (including cleaning, laundry and personal care products, and air fresheners) and found 133 distinct VOCs, with each product containing between 6–20 different VOCs and 24 of these VOCs were identified as toxic or hazardous under US federal law. The top three in terms of prevalence in the 25 tested products were all terpenoid species: limonene (in 23 products), α‐pinene (in 20), and β‐pinene (in 20). The concentrations of these three species in ~300 homes during the RIOPA (the Relationship of Indoor, Outdoor, and Personal Air) study were all found to be dominated by indoor sources.^9^ It is likely that cleaning product emissions contribute to the observed indoor concentrations of these VOCs.

When fragranced products are used, the individual components are released into the gas phase. Once released, they can then undergo oxidation by hydroxyl (OH) radicals, nitrate (NO_3_) radicals, and ozone (O_3_) to form a range of secondary pollutants including carbonyls, organic acids, particulate matter, organic nitrates, and peroxide species.^8^, ^10^, ^11^ Many of these secondary pollutants have been shown to be harmful to health.^12^, ^13^, ^14^ For instance, relatively high concentrations of formaldehyde (66 μg/m^3^, ~53 ppb) were found in a home with high limonene concentrations (~800 μg/m^3^, ~142 ppb), owing to very frequent use of cleaning products.^15^ For a similar home with much lower cleaning product use and limonene concentrations (~80 μg/m^3^, ~14 ppb), the formaldehyde concentration was also much lower (33 μg/m^3^, ~26 ppb). Formaldehyde is a known carcinogen and the World Health Organization (WHO) guidelines suggest a concentration of 100 μg/m^3^ should not be exceeded for more than 30 min per day.^16^ It is therefore important to understand the chemistry that results when cleaning products are used, in order to identify the conditions under which harmful pollutants may accumulate.

The rate at which the chemical components within a cleaning product will be transformed into secondary pollutants, will depend on the reactivity of the individual components with different oxidants and the consequent chemical reactions of the breakdown products. For instance, limonene, α‐pinene, and β‐pinene can all react with O_3_, but in doing so, form OH in varying yields (see Table 1). These terpenes also react with OH. Therefore, terpenes can be net OH radical sinks or sources, depending on the balance between these two processes. This point is important as OH will oxidize numerous VOCs indoors, so terpene chemistry following cleaning could lead to a much wider range of indoor chemistry than might be expected. Table 1 shows the rate coefficients for the reactions of α‐ and β‐pinene and limonene with OH, NO_3,_ and O_3_, as well as the yield of OH formation from the ozonolysis reactions. The final column shows an estimate of the ratio of the OH production to loss rate.

**TABLE 1:** Rate coefficients for the reactions of limonene, α‐pinene, and β‐pinene with OH, NO_3,_ and O_3_ at 298 K

Terpene	Rate coefficient cm^3^ molecule^−1^ s^−1^			OH yield	OH rate production/loss
	k(OH)	k(O_3_)	k(NO_3_)		
α‐pinene	5.3 × 10^−11^	9.4 × 10^−17^	6.2 × 10^−12^	0.80	1.42 × 10^−6^
β‐pinene	7.9 × 10^−11^	1.9 × 10^−17^	2.5 × 10^−12^	0.35	8.42 × 10^−8^
limonene	1.6 × 10^−10^	2.1 × 10^−16^	1.2 × 10^−11^	0.87	1.14 × 10^−6^

Note

Also shown is the yield of OH formed from the reactions between the terpenes and ozone. The final column shows an estimate of the ratio of OH production: loss calculated as {k(O_3_) × OH yield/k(OH)}. All rate coefficients and OH yields are from the MCM given the model described in this paper uses this reaction mechanism and are based on experimental data.^44^

Table 1 shows that limonene reacts more quickly with all three oxidants compared to the other two terpenoids. However, given the balance between formation of OH through ozonolysis and loss through reaction with OH, α‐pinene becomes marginally more important for net OH formation indoors. Depending on the mixtures of these species indoors, one might expect quite different oxidation chemistry and hence composition of indoor air.

**FIGURE 1:**
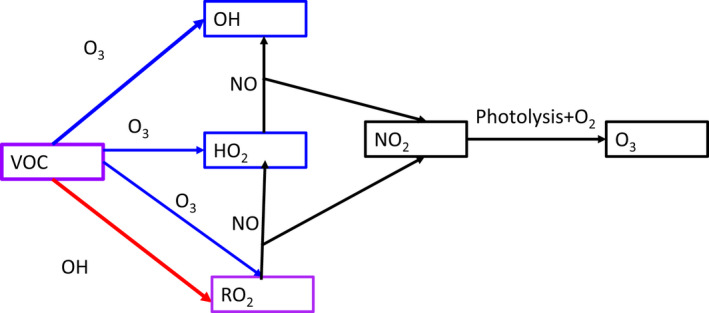
Connections between key indoor air species indoors, where HO_2_, hydroperoxy radical; NO, nitric oxide; NO_2_, nitrogen dioxide; O_3_, ozone; OH, hydroxy radical; RO_2_, generic term for organic peroxy radicals; VOC, volatile organic compounds

There are also connections between these oxidation processes as shown in Figure 1. When VOCs react with OH radicals, they can form peroxy (RO_2_ radicals). Peroxy radicals can then react with NO indoors (e.g., from cooking or outdoors) to make NO_2_, which can then be photolyzed at wavelengths below 420 nm to produce ozone. So OH can react with VOCs to make O_3_ and O_3_ can react with VOCs containing carbon‐carbon double‐bonds to make OH, as well as peroxy radicals.^17^ The presence of one oxidant will therefore likely produce the other and both will be associated with mixtures of radical species.

This paper uses a detailed chemical model for indoor air to investigate how the formulation of a cleaning product can impact the resulting indoor air chemistry. By varying the proportions of α‐pinene, β‐pinene, and limonene in a cleaning product, the different oxidation routes that arise can be investigated, as well as the impact on the indoor air chemistry. This study aims to identify the components that lead to the most harmful product mixtures, in order to make recommendations about how formulations might be modified to improve future indoor air quality. Although this work focuses on cleaning products, the results are broadly applicable to any product containing mixtures of similar species.

## 2 METHOD

### 2.1 Model description

The modeling simulations described in this paper have been carried out using the INdoor Detailed Chemical Model (INDCM) developed by Carslaw et al.^18^ This zero‐dimensional box model assumes a well‐mixed environment and bases its chemical mechanism on the Master Chemical Mechanism^19^, ^20^, ^21^, ^22^, which considers the degradation of common atmospheric VOCs through reactions with OH, NO_3_, O_3_ and by photolysis where relevant. Additional reactions are included to describe deposition onto indoor surfaces, indoor emissions, and exchange with outdoor air. The INDCM also considers photolysis reactions by indoor artificial lighting and attenuated outdoor light.^10^, ^18^

The concentration of each species in the INDCM is calculated according to Equation (1):

(1) $$\frac{dC_{i}}{\mathrm{dt}}=‐V_{d}\;\left(\frac{A}{V}\right)C_{i}+\lambda_{r}\;fC_{o}‐\lambda_{r}\;C_{i}+\frac{Q_{i}}{V}+\sum_{j=1}^{n}R_{\mathrm{ij}}$$

where *C*_i_ (*C*_o_) is the indoor (outdoor) concentration of species *i* (molecule cm^−3^), *V*_d_ its deposition velocity (cm s^−1^), *A* the surface area indoors (cm^2^), *V* the volume of air in the indoor environment (cm^3^), *λ*_r_ the air exchange rate with outdoors (s^−1^), *f* the building filtration factor, *Q*_i_/*V* the indoor emission rate for species *i* (molecule cm^−3^ s^−1^) and *R_ij_* the reaction rate between species *i* and *j* (molecule cm^−3^ s^−1^). For simplicity and in the absence of comprehensive measurements, we assume that *f* is 1 for all species, and that those that ingress from outdoors do so without being lost in the building envelope, for example, through deposition.

For the current work, the latest version of the Master Chemical Mechanism has been used (MCM v.3.3.1), which contains significant improvements to the OH and HO_2_ cycling in the isoprene degradation scheme compared to earlier versions.^23^ The representation of gas‐to‐particle reactions has also been improved in the INDCM for this work, with new gas‐to‐particle reactions added for α‐ and β‐pinene, and a more comprehensive representation for limonene. In order to make this improvement, the chemical degradation schemes for all three species were examined, and oxidation products with five or more C atoms that could potentially condense to form particles were identified. Short‐lived species such as radicals were ignored, as it was assumed that they were unlikely to contribute to particle mass given their lifetimes. This process identified 571 terpene oxidation species that had the potential to form particles.

The theory of Pankow^24^ was then used to define the gas‐to‐particle partitioning for the 571 oxidation species, assuming a dynamic equilibrium between the gas phase and condensed organic phase of each species.^25^ The partitioning coefficient for each species, *K*_p_, can be defined as shown in Equation 2:

(2) $$K_{p}=\frac{7.501RT}{{\;\mathrm{MW}}_{\mathrm{om}}10^{9\;}\gamma_{\mathrm{om}\;\;}V_{p}}$$

where the units of *K*_p_ are m^3^ µg^−1^, *R* is the ideal gas constant (8.314 J K^−1^ mol^−1^), *T* is the temperature (K), MW_om_ is the mean molecular weight of the absorbing particulate organic material (g mol^−1^), *V*_p_ is the liquid vapor pressure of the species (Torr), and *γ*_om_ is the activity coefficient of the species in the condensed organic phase. The aerosol is assumed to be well‐mixed, so the value of *γ*_om_ is set to unity.^25^, ^26^ The initial value of MW_om_ was assumed to be 120 g mol^−1^,^27^ but as the model run proceeds, this value is constantly recalculated, accounting for the molecular weights and proportions of each individual component of the overall particle mass. For instance, during the limonene only (LIM) simulation (see Table 2), the value ranged between 117 and 124 g mol^−1^.

**TABLE 2:** Description of the model simulations carried out for this work

Name	Description
Background	No cleaning activities
AP	α‐pinene only
BP	β‐pinene only
LIM	Limonene only
APBP	50:50 mix of α‐ and β‐pinene
LIMAP	50:50 mix of α‐pinene and limonene
LIMBP	50:50 mix of β‐pinene and limonene
kO_3_	The three terpenes were included with their concentrations in proportion to their rate coefficient for reaction with O_3_, k(O_3_), such that the product of k(O_3_) and the terpene concentration was the same for each of the 3 terpenes
kOH	The three terpenes were included with their concentrations in proportion to their rate coefficient for reaction with OH, k(OH), such that the product of k(OH) and the terpene concentration was the same for each of the 3 terpenes

A range of *V*_p_ estimation methods are available and these have been recently reviewed by Kruza et al.^28^ This research highlighted that the choice of vapor pressure method has a large impact on the predicted particle concentrations. Based on this work, the vapor pressures were calculated in the current work using the method proposed by Nannoolal et al.^29^ The calculation of vapor pressures requires an estimation of boiling points, which was carried out following the method of Nannoolal et al.^30^ These values can be calculated and downloaded online using UManSysProp^31^, ^32^ at this link http://umansysprop.seaes.manchester.ac.uk/tool/vapour_pressure.

Partitioning can be represented as a dynamic balance between absorption and desorption as presented in Equation (3).^25^, ^26^ A temperature and species independent value of 6.2 × 10^−3^ m^3^ µg^−1^ s^−1^ was used for *k*_on_, as sensitivity tests have shown that the predictions of particle mass are fairly insensitive to this value.^25^, ^26^ The value of *k*_off_ can then be found using Equation (3), assuming equilibrium conditions exist.

(3) $$K_{p}=\frac{k_{\mathrm{on}}}{k_{\mathrm{off}}}.$$

This equilibrium absorptive partitioning method will overestimate the condensed amount of all species, because there will be a finite kinetic uptake to the aerosol, that is, it will not be in equilibrium.^33^ However, the purpose of this work is to compare the relative amounts of particles formed for different formulations rather than the absolute concentrations and is sufficient for the current application. Note that the model does not treat size distribution of the classes and that the particles formed through chemistry are likely to exist in the fine and ultrafine fraction.^34^

### 2.2 Simulations

Singer et al.^8^ described cleaning with a general‐purpose cleaner that contained eight terpene hydrocarbon species, five terpene alcohols, and two other VOCs. During cleaning experiments, they measured the concentrations of these constituents for several hours following floor mopping or surface cleaning in a 50 m^3^ chamber with an air exchange rate (AER) of 0.5 h^−1^. For the hour immediately following cleaning and in the absence of ozone and averaged over three experiments, the total terpene hydrocarbon concentration was ~450 ppb (~2.5 mg/m^3^). Limonene and terpinolene were the dominant constituents in these experiments, both averaging around 190 ppb (~1.1 mg/m^3^). The MCM currently contains schemes for three of the measured terpene hydrocarbons, α‐ and β‐pinene, and limonene. A number of model runs were therefore carried out using these three species in varying proportions as a proxy for a typical mixture of terpene hydrocarbons that might be found in cleaning formulations (Table 2). For comparison, a run with no cleaning was also included in the simulations. For this run, there were only background concentrations of the terpene species that derived from exchange with outdoors. This exchange provided average concentrations of ~3 ppb (~17 μg/m^3^) of limonene, 200 ppt (~1.1 μg/m^3^) of α‐pinene, and 1 ppt (~0.01 μg/m^3^) of β‐pinene in the absence of indoor sources.

It was assumed that cleaning was carried out at 8:00 h in the morning and in a 50 m^3^ room. Cleaning was assumed to last for ~10 min and the emissions of the different terpenes were set such that the average total concentration of the terpene hydrocarbons in the hour following cleaning was similar to those reported by Singer et al.^8^. For example, this value ranged from 227 to 484 ppb for air exchange rates of 2 and 0.2 h^−1^, respectively. The temperature was assumed to be 296 K with a relative humidity of 45%, with an AER of 1 h^−1^.

The outdoor O_3_ (ozone), NO_2_ (nitrogen dioxide), NO (nitric oxide), and VOC (volatile organic carbon) concentrations were set to be typical for a European city in summer as described by Kruza and Carslaw.^35^ Outdoor O_3_, NO_2_, and NO mixing ratios are 49, 19, and 16 ppb, respectively, and PM_2.5_ concentrations are 17.5 μg/m^3^ averaged over 09:00–17:00 h. For the same time period, this leads to average indoor values of 8, 10, and 2 ppb and 14 μg/m^3^, respectively, in the absence of cleaning. Indoor VOC concentrations were taken from Sarwar et al.^36^ or Zhu et al.^37^ It is assumed that 3% of outdoor UV and 10% of outdoor visible light enters the building through the windows as described in Carslaw.^18^ The values used for indoor deposition velocities are described in detail in Carslaw et al.^10^

### 2.3 Sensitivity tests

Sensitivity runs were also carried out where the 9 simulation conditions described in Table 2 were repeated but with the AER first set to 0.2 and then to 2 h^−1^. Higher air exchange rates allow higher rates of ingress of outdoor air pollutants, while diluting indoor emissions more rapidly. Such conditions increase indoor ozone concentrations as more outdoor ozone can ingress. At lower air exchange rates, less outdoor ozone can ingress, but chemical reactions indoors effectively have more time to occur before removal by ventilation. A final set of sensitivity runs tested the effects of cleaning at 4 PM in the afternoon instead of the morning, when outdoor ozone concentrations and light transmission rates through windows are higher. For these sensitivity runs, the same terpene emission rates were used as for the baseline run, so assuming similar cleaning conditions.

## 3 RESULTS AND DISCUSSION

**FIGURE 2:**
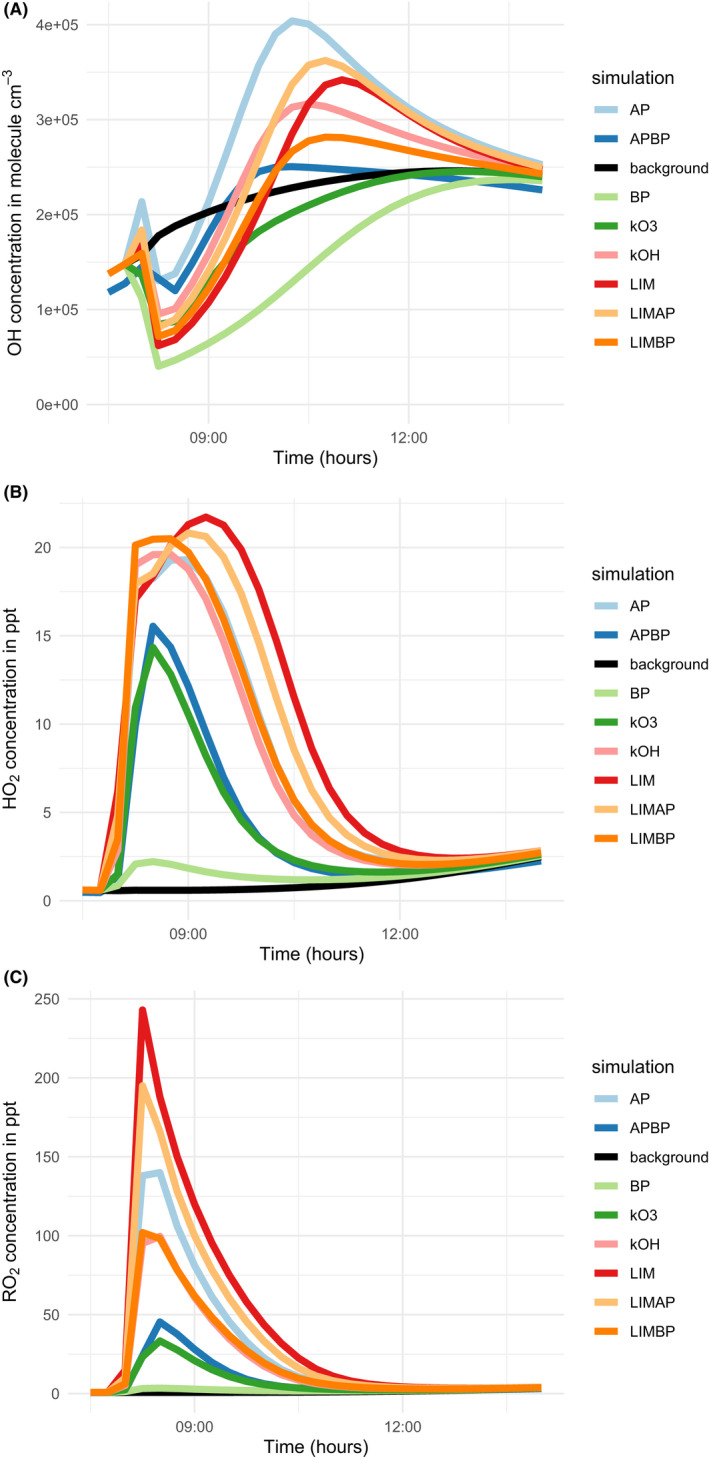
Concentrations or mixing ratios of (a) OH, (b) HO_2,_ and (c) RO_2_ radicals in the upper, middle, and lower plots, respectively, between 07:30 and 14:00 h during the different model simulations

The predicted hydroxy (OH), hydroperoxy (HO_2_), and organic peroxy (RO_2_) radical concentrations and mixing ratios are shown in Figure 2, which shows that the chemistry indoors is strongly affected by the terpene mixture. Beta‐pinene is the least efficient at producing radicals through its chemical reactions following cleaning and acts as a net sink for OH over the course of the model simulation. Pure limonene makes HO_2_ and RO_2_ radicals very efficiently, whereas pure α‐pinene is most efficient at making OH radicals. The OH is removed by all terpene mixtures when cleaning takes place and recovers at different rates depending on the rate coefficients and the consequential differential impacts on OH production presented and discussed in Table 1.

The 50:50 α‐pinene: β‐pinene mixture makes approximately half the peak OH concentration compared to the α‐pinene on its own, though β‐pinene has a less pronounced modifying effect on OH production from limonene. Adding β‐pinene to limonene reduces the RO_2_ mixing ratio significantly compared to limonene on its own and also reduces RO_2_ made from pure α‐pinene when added in equal proportion. Figure 2 also shows that there are sustained concentrations of radicals for several hours following cleaning, despite the cleaning‐related emissions only lasting for around 10 min. Most radical concentrations are back to no clean levels by between 14:00 and 15:00 h, around 6 h after cleaning.

**FIGURE 3:**
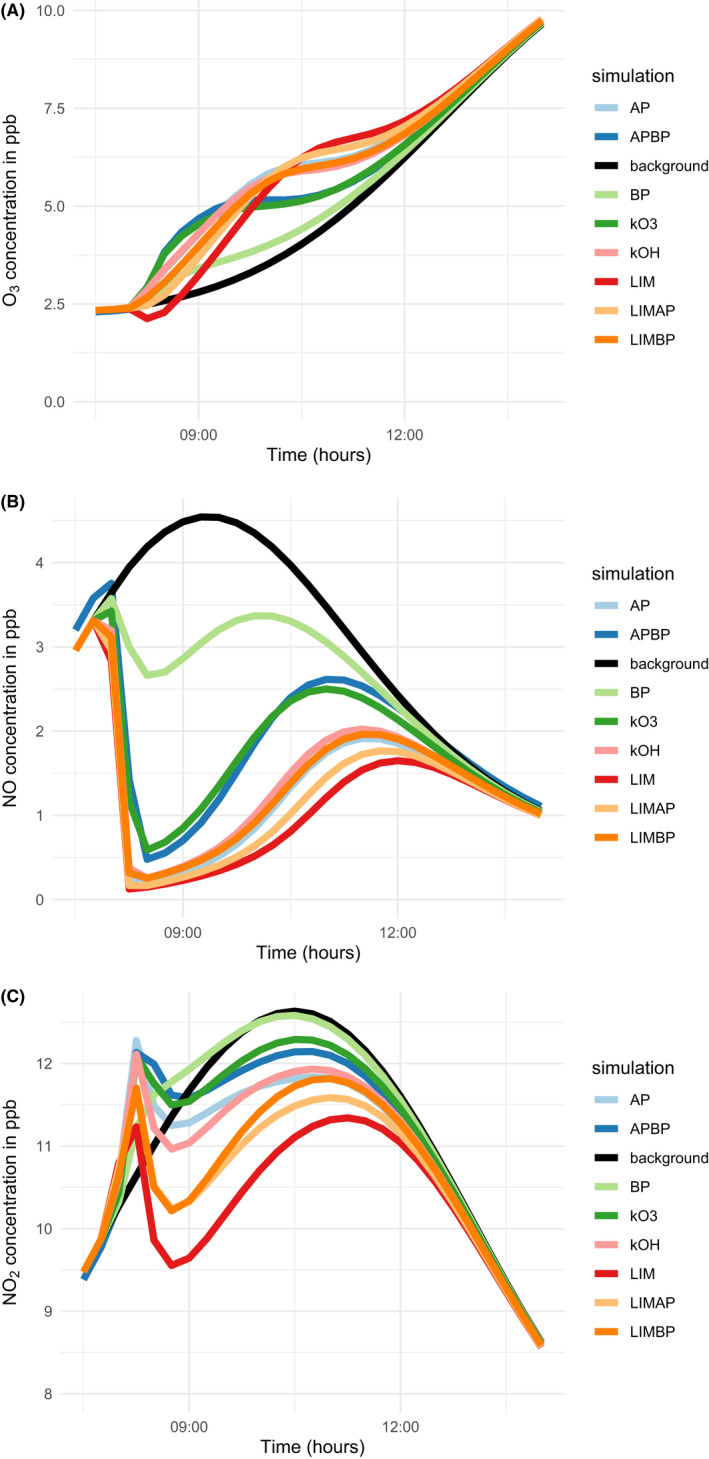
Concentrations of O_3_, NO, and NO_2_ in the upper, middle, and lower plots, respectively, between 07:30 and 14:00 h during the different model simulations

Figure 3 shows the mixing ratios for O_3_, NO, and NO_2_ during the different simulations. Ozone mixing ratios are higher for all of the cleaning simulations when compared to the simulation with no cleaning. This is because the high concentrations of RO_2_ and to a lesser extent, HO_2_ following cleaning activities can remove NO (reactions R1–R2), which would otherwise react with O_3_ such as shown in reaction R3:

(R1a) $${\mathrm{RO}}_{2}+\mathrm{NO}\to \mathrm{RO}+{\mathrm{NO}}_{2}$$

(R1b) $${\mathrm{RO}}_{2}+\mathrm{NO}\to {\mathrm{RNO}}_{3}$$

(R2) $${\mathrm{HO}}_{2}+\mathrm{NO}\to \mathrm{OH}+{\mathrm{NO}}_{2}$$

(R3) $$\mathrm{NO}+O_{3}\to {\mathrm{NO}}_{2}+O_{2}$$

Peroxyacetyl radicals (which are a subset of the organic peroxy radicals) react with NO_2_ to form peroxyacetyl nitrate (PAN) species, for example, for R4:

(R4) $${\mathrm{RCO}}_{3}+{\mathrm{NO}}_{2}\to {\mathrm{RCO}}_{3}{\mathrm{NO}}_{2.}$$

Therefore, the net effect of the high peroxy radical concentrations is to remove nitrogen oxides which allows ozone to accumulate relative to the case with no cleaning. The simulations which have the highest RO_2_ concentrations, namely pure limonene and the limonene/α‐pinene mixture, have the lowest NO concentrations (Figure 3b).

Reactions R1b and R4 show how the organic nitrates and PAN type species are formed from chemical reactions following cleaning. Aldehydes can be formed when alkoxy radicals (RO in reaction R1a) react with O_2_ to form HO_2_ and an aldehyde, for example, methoxy radicals react with O_2_ to form formaldehyde in R5:

(R5) $${\mathrm{CH}}_{3}O+O_{2}\to \mathrm{HCHO}+{\mathrm{HO}}_{2}$$

**FIGURE 4:**
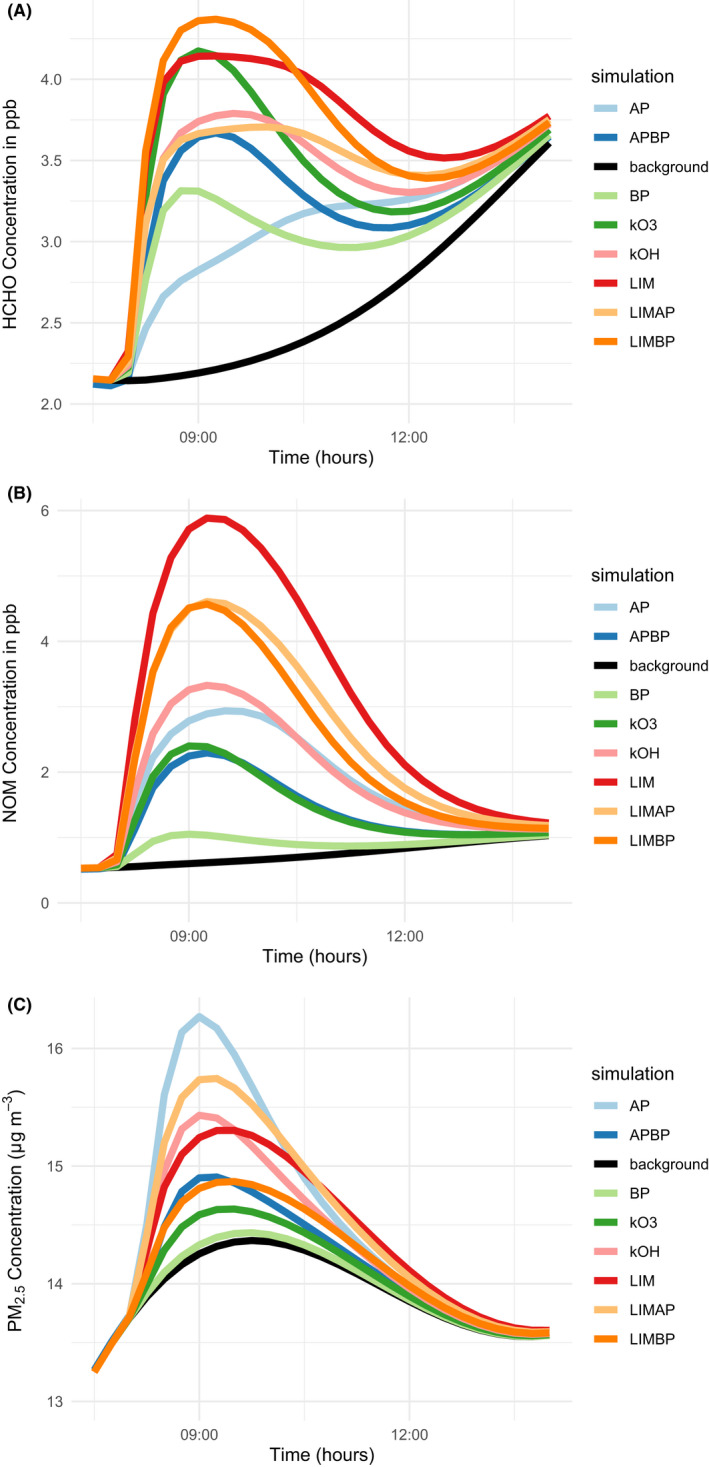
Concentrations of HCHO, nitrated organic material (NOM), and PM_2.5_ in the upper, middle, and lower plots, respectively, between 07:30 and 14:00 h during the different model simulations

Figure 4 shows the results from the simulations for formaldehyde (HCHO), nitrated organic material (includes the sum of organic nitrates, generic formula RNO_3_ and PANs, generic formula RCO_3_NO_2_), and fine particulate matter (PM_2.5_) formed through the chemistry. Given the adverse health effects of these species (e.g., Ref. [38, 39, 40, 41]) they serve as a proxy for the harmful species that can be formed through chemical reactions when cleaning products are used in the different formulations described here.

Limonene is the most efficient formaldehyde‐forming terpene on its own. Both the pure limonene and pure β‐pinene show a formaldehyde peak shortly after cleaning, while the α‐pinene concentration increases more gradually. A 50:50 mixture of limonene and β‐pinene becomes even more efficient at producing formaldehyde than the pure substances for the first part of the model simulation, even though the total terpene concentration is the same. These different profiles can be understood by reference to the rates of reaction for the different simulations.

For both the pure limonene and pure β‐pinene, the major route to formaldehyde formation is the decomposition of the alkoxy radical that results from oxidation by OH, followed by reaction with NO/RO_2,_ and finally, degradation of the alkoxy radical as described by the MCM protocol.^19^ These reactions are shown for β‐pinene (R6–R9):

(R6) $$\beta \;\text{‐ pinene}+\text{OH}\to {\text{BPINAO}}_{2}$$

(R7) $${\mathrm{BPINAO}}_{2}+\mathrm{NO}\to \mathrm{BPINAO}\;\left(76\%\right)$$

(R8) $${\mathrm{BPINAO}}_{2}+{\mathrm{RO}}_{2}\to \mathrm{BPINAO}\left(70\%\right)$$

(R9) $$\mathrm{BPINAO}\to \mathrm{HCHO}+\mathrm{nopinone}+{\mathrm{HO}}_{2}$$

and for limonene (R10–R13):

(R10) $$\mathrm{limonene}+\mathrm{OH}\to {\mathrm{LIMCO}}_{2}\left(37\%\right)$$

(R11) $${\mathrm{LIMCO}}_{2}+\mathrm{NO}\to \mathrm{LIMCO}\;\left(77\%\right)$$

(R12) $${\mathrm{LIMCO}}_{2}+{\mathrm{RO}}_{2}\to \mathrm{LIMCO}\;\left(70\%\right)$$

(R13) $$\text{LIMCO}\to 4\;\text{‐ acetyl ‐ l ‐ methylcyclohexene}+\text{HCHO}+{\text{HO}}_{2}$$

where BPINAO_2_ and LIMCO_2_ are the peroxy radicals formed following reaction of OH with β‐pinene and limonene, respectively, and BPINAO and LIMCO are the alkoxy radicals formed when the peroxy radicals react with NO or other peroxy radicals. For the pure mixtures, the rate of R9 just exceeds that of R13 about 1.5 h after cleaning (2.7 × 10^7^ molecule cm^−3^ s^−1^ cf. 2.6 × 10^7^ molecule cm^−3^). For the 50:50 mixture, these rates change to 2.1 × 10^7^ molecule cm^−3^ s^−1^ and 1.6 × 10^7^ molecule cm^−3^, giving a total HCHO production rate of 3.7 × 10^7^ molecule cm^−3^ s^−1^, exceeding that of the pure compounds. Figures 2, 3, 4 show other differences between these scenarios at the same time. Ozone mixing ratios for the β‐pinene/limonene mixture (11.2 ppb) are 17% higher than for the pure limonene and 25% higher than the pure β‐pinene (8.9 ppb) scenarios (Figure 3a). However, the OH concentration for the mixture at this time is 4.9 × 10^5^ molecule cm^−3^, 20% higher than for pure limonene, but 75% higher than for pure β‐pinene. So R9 becomes relatively more important in the mixture compared to R13, as would be expected from the fact that the rate coefficient for ozonolysis of limonene is 10× faster than that for beta‐pinene, whereas the hydroxyl radical reaction with limonene is only 2× faster than that for beta‐pinene (Table 1). This result shows that there are intricate chemical links between these species and the impacts of different mixtures on the indoor air composition need to be carefully considered.

Pure limonene and its mixtures with the pinenes are by far the most efficient at forming nitrated organic material. This links in part to their propensity to form RO_2_ efficiently through R1b and R4. Pure β‐pinene is again very inefficient at producing this nitrated material. Finally, pure α‐pinene is the most efficient at producing particulate matter from cleaning, while pure β‐pinene is the least efficient. These differences arise because of the different structures of the terpene species and in particular, the position of the carbon‐carbon double bond. The highest OH concentrations existed for the pure α‐pinene mixture (Figure 2) as expected from Table 1, and ozone concentrations were also relatively high for this terpene (Figure 3). The higher oxidant concentrations facilitate increased particle formation for these simulations.

The mixtures that were simulated based on rate coefficients for reaction with ozone (the kO_3_ mixture) and hydroxyl radicals (the kOH mixture) reflect the proportions of the parent terpenes used. For the kO_3_ mixture, 78, 16, and 7% of the mixture was β‐pinene, α‐pinene, and limonene, respectively. For the kOH mixture, the same values were 33, 51, and 16%. In Figures 2, 3, 4, the kO_3_ mixture tends to most closely follow the α‐pinene/β‐pinene mixture or pure β‐pinene. The kOH mixture demonstrates slightly more complex behavior, perhaps owing to the less divergent proportions of each terpene compared to the kO_3_ mixture. However, it most often behaves in a similar manner to pure α‐pinene.

Table 3 shows the results of the sensitivity studies. Maximum OH concentrations are noted for the pure α‐pinene simulations except for 0.2 h^−1^, when OH is removed for all simulations relative to no cleaning. For HO_2_ and RO_2_, maximum mixing ratios are observed for pure limonene for baseline conditions and for the limonene/α‐pinene mixture when the AER is 2 h^−1^. When the air exchange rate is only 0.2 h^−1^ and for afternoon cleaning, HO_2_ peaks for the kO_3_ mixture. RO_2_ mixing ratios peak for the limonene/α‐pinene mixture when AER is 0.2 h^−1^ and for pure α‐pinene alone for the afternoon cleaning. RO_2_ (and HO_2_ to a lesser extent) concentrations tend to be controlled by reaction with NO (see R1).

**TABLE 3:** Average concentrations or mixing ratios for the 3‐h period following cleaning for the nine different model simulations (see Table 2) for baseline conditions, with air exchange rates of 0.2 and 2 h^−1^, respectively, and when cleaning starts 4 PM instead of 8 AM

	No clean	LIM	AP	BP	kOH	kO_3_	APBP	LIMAP	LIMBP
OH/10^5^ molecule cm^−3^									
Baseline	2.1	1.8	**2.8**	0.94	2.2	1.6	2.0	2.2	1.8
AER = 2 h^−1^	1.9	2.9	**3.8**	1.4	3.0	2.2	2.8	3.3	2.7
AER = 0.2 h^−1^	**1.1**	0.23	0.36	0.26	0.27	0.24	0.31	0.25	0.22
Afternoon	1.9	2.3	**3.0**	1.1	2.2	1.5	2.0	2.6	1.9
HO_2_/ppt									
Baseline	0.6	**16.5**	12.9	1.5	12.3	6.6	7.1	15.0	13.3
AER = 2 h^−1^	0.3	9.0	7.6	0.6	7.0	2.9	4.9	**9.4**	7.7
AER = 0.2 h^−1^	4.1	8.1	7.9	6.5	9.3	**10.7**	10.0	7.8	9.8
Afternoon	2.2	10.3	9.4	8.9	10.3	**11.2**	10.3	9.8	11.1
RO_2_/ppt									
Baseline	0.8	**88.1**	55.7	2.3	40.8	13.2	16.4	72.4	42.4
AER = 2 h^−1^	0.4	32.1	25.9	0.9	19.1	5.3	11.0	**36.1**	20.6
AER = 0.2 h^−1^	5.8	121.8	120.5	11.3	87.1	36.6	44.7	**138.3**	86.2
Afternoon	3.4	313.5	**499.2**	20.0	346.7	111.0	249.3	284.4	304.0
HCHO/ppb									
Baseline	2.2	3.9	2.9	3.0	3.5	3.7	3.3	3.5	**4.0**
AER = 2 h^−1^	2.9	4.2	3.5	3.3	3.9	3.9	3.9	4.0	**4.3**
AER = 0.2 h^−1^	1.1	1.3	1.0	**1.5**	1.3	1.4	1.3	1.2	1.4
Afternoon	4.4	5.9	5.0	5.7	5.7	5.9	5.7	5.5	**6.3**
O_3_/ppb									
Baseline	3.2	4.1	**4.7**	3.6	4.6	4.4	4.5	4.4	4.5
AER = 2 h^−1^	4.7	6.1	6.1	4.9	6.1	5.6	5.9	**6.2**	6.1
AER = 0.2 h^−1^	**1.4**	0.7	1.0	1.4	1.0	1.2	1.2	0.8	0.9
Afternoon	11.1	10.1	10.6	11.4	10.7	**11.3**	11.2	10.7	10.2
NO/ppb									
Baseline	**4.2**	0.6	0.9	3.1	1.0	1.6	1.6	0.7	0.9
AER = 2 h^−1^	**5.8**	2.7	2.9	5.3	3.0	3.8	3.4	2.6	2.9
AER = 0.2 h^−1^	**0.6**	0.1	0.1	0.4	0.1	0.2	0.2	0.1	0.1
Afternoon	**0.9**	0.2	0.2	0.5	0.2	0.3	0.2	0.2	0.2
NO_2_/ppb									
Baseline	11.8	10.5	11.6	**12.0**	11.5	11.8	11.8	11.0	11.1
AER = 2 h^−1^	17.7	17.3	18.0	17.8	17.9	18.1	**18.1**	17.6	17.7
AER = 0.2h^−1^	**3.1**	2.5	2.9	3.1	2.8	2.9	3.0	2.6	2.6
Afternoon	**8.9**	6.5	7.7	8.6	7.5	8.0	8.1	7.1	6.8
NOM/ppb									
Baseline	0.6	**4.6**	2.4	0.9	2.6	1.8	1.8	3.7	3.5
AER = 2 h^−1^	0.7	**3.7**	2.0	0.8	2.1	1.4	1.6	3.1	2.9
AER = 0.2 h^−1^	0.6	**1.7**	1.0	0.7	1.2	1.0	0.9	1.5	1.5
Afternoon	1.1	**5.4**	3.0	1.6	3.6	2.6	2.5	4.2	5.1
PM/μg m^−3^									
Baseline	14.2	14.9	**15.3**	14.2	14.9	14.4	14.6	15.2	14.6
AER = 2 h^−1^	16.8	17.3	17.3	16.8	17.1	16.8	16.9	**17.4**	17.0
AER = 0.2 h^−1^	6.9	7.1	**7.6**	6.9	7.3	7.1	7.2	7.3	7.1
Afternoon	14.8	16.5	**19.8**	14.9	17.7	15.7	17.2	17.0	16.2

Note

Numbers denoted in bold on each row are the maximum values over all formulations for each scenario.

Maximum HCHO concentrations are observed for the limonene/β‐pinene mixture, except when AER is 0.2 h^−1^, when the pure β‐pinene scenario produces most. For O_3_, the maximum average mixing ratios are observed for pure α‐pinene for baseline conditions, and the limonene/α‐pinene mixture when AER is 2 h^−1^. For an AER of 0.2 h^−1^, ozone is removed relative to the no clean scenario for all simulations. For afternoon cleaning, the kO_3_ mixture is most efficient at producing ozone.

Nitric oxide is removed by all mixtures and for all of the simulation conditions, while NO_2_ peaks for the α‐pinene/β‐pinene mixture when AER is 2 h^−1^. For AER of 0.2 h^−1^ and afternoon cleaning, all formulations remove NO_2_ relative to the non‐clean scenario. The nitrated organics peak for the pure α‐pinene simulations for all of the scenarios. PM concentrations peak for the α‐pinene conditions for all runs except when AER is 2 h^−1^, when they peak for the mixture of limonene and α‐pinene.

The main difference between the morning and afternoon cleaning scenarios are the ozone and NO mixing ratios, which are a factor of 3–4 higher and lower, respectively, in the afternoon compared to the morning under baseline conditions (Table 2). The OH concentrations are broadly similar. Ozone oxidation is therefore relatively more important in the afternoon. The maximum RO_2_ mixing ratios are much higher than in the morning, reflecting the enhanced chemistry with additional ozone, although pure β‐pinene produces low RO_2_. However, unlike the morning cleaning scenario, the combination of β‐pinene and limonene produces almost as much RO_2_ as limonene on its own. The highest HCHO mixing ratios are again derived from the 50:50 β‐pinene/limonene mixture, with peak values about double that observed in the morning.

Focusing on the harmful secondary pollutants, afternoon cleaning tends to produce higher concentrations than cleaning in the morning. Increasing the AER from 1 h^−1^ (baseline conditions) to 2 h^−1^, also produces more PM and HCHO for all scenarios. Although the PM and HCHO formed indoors are diluted under this higher ventilation rate, there is also more ozone available to initiate chemistry and form these pollutants indoors. In addition, there is more transport of these species indoor from outdoors. For PM, although concentrations increase overall with air exchange rate, the composition will be increasingly influenced by PM outdoors as air exchange rate increases.^10^, ^42^ For the production of nitrated organic material, increasing AER to 2 h^−1^ reduces mixing ratios relative to baseline conditions. This is because higher outdoor NO suppresses the RO_2_ mixing ratio relative to the baseline conditions, such that the production rate of this material is lower overall than under baseline conditions.

We also investigated the impact of changing temperatures for two of our formulations, pure limonene and the 50:50 mixture of alpha‐ and beta‐pinene. Predicted concentrations of radicals were most sensitive to these changes, particularly for increased temperatures. When we increased the temperature to 306 K, OH concentrations increased by 34 and 47%, respectively, for the 100% limonene and 50:50 pinene mixture relative to 296 K. Reducing the temperature to 286K produced OH concentration decreases of 22 and 17% for pure limonene and the pinene mixture, respectively. However, in terms of changes to the potentially harmful secondary species, these were generally less than 10%. The exception is the predicted organic nitrate concentration, which increased by 31 and 12% for the pure limonene and 50:50 pinene formulations, respectively, when the temperature was increased to 306 K. We may therefore expect higher concentration of organic nitrates during cleaning at higher temperatures.

## 4 CONCLUSIONS

This study has shown that different combinations of terpenes, either singly or in mixtures, can give rise to a variety of reaction products. The results have shown that pure β‐pinene is generally very inefficient at producing radicals and potentially harmful secondary pollutants. Pure limonene and α‐pinene tend to be most efficient at radical production. While pure α‐pinene is most efficient at producing particulate matter, limonene is most efficient at forming nitrated organic material. However, the 50:50 β‐pinene: limonene mixture is best at making formaldehyde.

Clearly then, the composition of the formulation used for cleaning or other indoor products can have an important impact on the ongoing chemistry indoors. On the face of it, replacing α‐pinene and limonene with β‐pinene could reduce the production rate of secondary pollutants, though the formaldehyde results show that the resulting chemistry can be complex. What is also needed, is a more comprehensive understanding of the full product mixture in cleaning products, so that comprehensive assessments can be made of the likely impact of different formulations on harmful pollutant production indoors.

Finally, there exist relatively few studies at the moment that assess the health effects of oxidation products formed from terpene oxidation (such as those presented in Ref. [43]). This absence of information makes assessing the relative health benefits of including or excluding different cleaning product components challenging. In fact, even deciding if an increase in NO_2_ is offset by a decrease in PM concentration and the size of such an offset needed to accrue a benefit, is difficult with current knowledge.
